# “The plants have axé”: investigating the use of plants in Afro-Brazilian religions of Santa Catarina Island

**DOI:** 10.1186/s13002-020-00372-6

**Published:** 2020-04-25

**Authors:** Tiago Santos Pagnocca, Sofia Zank, Natalia Hanazaki

**Affiliations:** Laboratory of Human Ecology and Ethnobotany (ECOHE), Department of Ecology and Zoology, Federal University of Santa Catarina (UFSC), Campus Universitário Reitor João David Ferreira Lima, s/n, Florianópolis, SC 88040-900 Brazil

**Keywords:** Ethnobotany, Medicinal plants, Candomblé, Umbanda, Ritual de Almas e Angola

## Abstract

**Background:**

Cultural and religious practices of African origin have decisively influenced traditional health practices in the Americas since the African diaspora. Plants are core elements in the religions of African origin. Compared with other parts of Brazil where the Afro-Brazilian presence is widely recognized, in Southern Brazil, these cultural practices are often socially invisible. Yet, there are several terreiros of three Afro-Brazilian religions: Candomblé, Umbanda, and Ritual deAlmas e Angola. We hypothesize that the importance of plants in Afro-Brazilian religions is linked not only to spiritual and magical issues but also to the medicinal properties of these plants. We seek to answer the following questions: (a) Which plants are used in the terreiros and what are their indications for use?; (b) Are there plants that stand out culturally in these religious groups?; and (c) What is the importance of the adaptive maintenance and replacement process in the use of plants in these religions, considering the Neotropical and African plants?

**Methods:**

We performed a census of the existing terreiros on the Island of Santa Catarina to collect information on the knowledge and use of plants. In all terreiros that consented to participate in the research, we collected data through semi-structured interviews, guided tours for plant collection, and participant observation. We identified the botanical species through expert consultations and botanical literature.

**Results:**

We interviewed 27 spiritual leaders, who cited 93 plants belonging to 86 botanical species. We identified 14 categories of use, with emphasis on liturgical ritual use (59%), general and unspecified diseases (32%), and digestive diseases (27%). In most liturgics uses, direct contact between plant and patient occurs, as in the case of bathing and the cleansing use of smoke. Sixteen plants were cited in all terreiros, configuring a set of species that can be considered as culturally important plants for these religious groups.

**Conclusions:**

These groups have extensive knowledge about a highly consensual set of therapeutic plants that should be further investigated pharmacologically to understand the effect of their external use. Also, we emphasize the importance of recognizing and valuing this ancestral Afro-Brazilian knowledge and learning also from these people about their broader vision of health which also adds more spirituality in health care.

## Introduction

The African diaspora, a sociocultural and historical phenomenon, characterized by the forced immigration of the African population to countries that adopted slave labor, had a decisive influence on traditional health practices on the American continent. In the process, knowledge linked to health and the religious beliefs and traditions of African peoples has been combined with the knowledge of indigenous and European peoples who already inhabited this continent [1, 2]. The relationships between African descendants and plants are addressed in various ethnobotanical studies related to: (a) the cultural contributions from medicinal and ritualistic practices [1–10]; (b) the exchange of plant species between the American and African continents and how these species became part of the landscape and local culture [5, 11–13]; and (c) the influence of plant knowledge on the identity of Afro-Brazilian religions [4, 14, 15].

Africans have been present in Brazil since the early days of colonization in the sixteenth century. During this period, it is estimated that about 4.9 million people were brought as slaves to the Brazilian territory [16]. They were taken from various African regions and belonged to different ethnic groups; such as the Bantu speaking people from Angola, Congo, and Mozambique [1, 17]; and groups of Sudanese, from West Africa, from territories currently called Nigeria, Benin (ex-Dahomey), and Togo [18, 19]. In the process of adaptation to the new territory, slaves became familiar with local plants, learning and adapting their knowledge of medicinal and magical properties and remodeling their medicinal-ritualistic system [20]. In this context, Medeiros et al. [21] argued that immigration provides two main processes for adapting knowledge: (1) replacement to the new flora of the host country and (2) maintenance through use and acquisition of the original flora from migrants’ home countries.

Plants are key elements in the Afro-Brazilian religions, in which health is understood in a comprehensive manner, considering physical and spiritual aspects [3, 22]. In addition to direct use as a medicinal resource, as a remedy to cure physical illnesses, plants are also used in a liturgical and ritualistic way, through bathing, smoke cleansing, and other uses, as already described in ethnopharmacological studies [23]. It is important to assume that medicinal plants are frequently used with a ritualistic component, and rituals do play a role in the well-being of individuals and their cultural identity [24].

Currently, there are several religions of African origin in Brazil, which have been recreated and adapted over the centuries [25], even in the face of prejudice and racism. In southern Brazil, black people and their cultural and religious practices still suffer from a process of social invisibility, as a result of both historical racial prejudice [26, 27], and of the historical immigration characteristics of southern Brazil that especially value the European migrant identity. This is the first study to use an ethnobotany approach to address the plant uses in Afro-Brazilian religions in this region. In this context, we emphasize the importance of this research, aimed at registering and valuing the knowledge and practices of Afro-Brazilians, in order to strengthen the black identity and the social recognition of these groups in South Brazil.

On the island of Santa Catarina, in southern Brazil, three Afro-Brazilian religions are practiced: Candomblé, Umbanda, and Ritual de Almas e Angola (ritual of Souls and Angola); presenting a promising context for studies on their relationship with plants in their religious contexts. We hypothesize that the importance of plants in Afro-Brazilian religions is linked not only to spiritual and magical issues but also to the medicinal properties of these plants. In this way, we investigate the use of plants in these Afro-Brazilian religions, seeking to understand their importance in relation to their cultural, magical, and medicinal properties. We seek to answer the following questions: (a) Which plants are used in the terreiros and what are their indications for use including liturgical and physical illnesses’ spheres?; (b) Are there plants that stand out culturally in these religious groups?; (c) What is the importance of the adaptive maintenance and replacement process in the use of plants in these religions, considering the Neotropical and African plants used for liturgic or physical illnesses?

## The religions of African origin in Brazil

The ritualistic and religious activities of Afro-Brazilians occurred underground for many years, and gradually evolved into more organized gatherings. These ritual practices were called: calundú [28], drumming; and batuquejê, typified by collective dances, songs, percussion music, the invocation of spirits, possession sessions, divination, and magic rituals. These “societies,” formed during the 17th and 18th centuries, are considered the antecedents of the candomblé of the 19th century [20, 28]. In order to unite and strengthen themselves, slaves began forming the “saint families” (famílias de santo), composed of slaves from the same nation of origin. This led to the first terreiros, where Africans and their descendants gathered and established links based on ties of religious kinship [29]. The terreiros are the physical spaces where the religious ceremonies and their ritualistic activities take place, such as offerings, sharing the stories and fables of the saints, initiations, and where the conviviality of the familia de santo occurs.

Candomblé emerged as a result of the maturing of the calundús, which began to gather groups of blacks in an organized manner and thus solidified and gradually developed a religious structure. In principle, rituals were restricted to domestic spaces and took place periodically, but with the abolition of slavery in 1888 and the proclamation of the Republic, they began to establish themselves as organized congregations in extra-domestic spaces. This started an Afro-Brazilian religious community that later consolidated as the Candomblé religion [1, 28]. One of the main symbolic elements within Candomblé is the worship of Orixás (deities associated with the elements of nature). There are specific offerings for each Orixá and selected plants that are used in initiation rituals, baths, and other “obligations” [17]. In Candomblé, the Orixá known as the deity of the leaves is Ossaim, the owner of the forests and holder of all the wisdom and knowledge of the vegetable kingdom [17]. According to the African legends, Ossaim lives in the woods, where he learned all the secrets of the magic of the herbs.

Umbanda is a genuinely Brazilian religion, structured under the influence of the Iberian, Amerindian, and African cultures. According to Saraceni [30], Umbanda emerged as a branch of Candomblé around the mid-19th century, and by the beginning of the 20th century was so powerful that it had spread throughout Brazil [31, 32]. It is a Spiritist religion, strongly influenced by Kardecism (created in France by Allan Kardec and adopted in Brazil since the middle of the last century [30]).

In the 1940s, thirty years after its emergence, there were numerous branches of Umbanda, combining different ritual practices. During this period, the Umbanda de Almas e Angola was added to this ritualistic plurality, mixing Umbanda concepts with Candomblé practices. The Ritual de Almas e Angola practiced in Santa Catarina can be defined as a branch of Umbanda, but with particularities that bring it very close to Candomblé [33].

One of the basic foundations of these three Afro-Brazilian religions is the use of plants in their liturgical rituals since they are carriers of axé, a primordial element for the accomplishment of ritual and religious works [34]. In these three ritual practices, plants are understood as essential parts of the healing practices. It is believed that plants can influence the spiritual plane and energetic layers that make up the aura of living beings, through an exchange of energetic fields. They can neutralize certain energies and enhance others, having a balancing effect.

## Methods

### Study area

The Island of Santa Catarina is located in the Southern region of Brazil, in the State of Santa Catarina. It is 54 km long, 18 km wide and has a territorial area of 424.40 km^2^, of which approximately 29 km^2^ are rivers and lagoons, with a great diversity of vegetation, hydrography, topography, and geology [35]. The island has two main vegetation formations in different successional stages: dense rainforest and coastal vegetation, which includes mangroves and restingas (sand dune vegetation) [36]. The island harbors the major part of the city of Florianópolis, the capital of the State of Santa Catarina. The population is estimated at 477,798 inhabitants, comprising approximately 14% blacks and 86% non-blacks [37].

At the time of European colonization of the island (mid-17th century), the area was inhabited by Amerindians from the Tupi-Guarani group, known as Carijós, whose subsistence was based on agriculture, fishing, and collecting mollusks [38]. The Portuguese colonization recognized the island as a strategic port for expeditions to the South Atlantic, with a strategic role in the defense of Brazil’s southern limits [39]. From the mid-18th century, immigrants from Azores, Madeira, and continental Portugal settled on the Santa Catarina coast, expanding agriculture and fishing practices [40, 41].

African slaves were present on the Island since 1738, where they were forced to work on fortresses construction and whaling [42]. After the arrival of the Azorean immigrants, the number of African slaves also increased to work in the plantations, mills, and performing domestic services. Slaves always played roles that helped to build the city’s economic and social history [43]. The arrival of black people on the Santa Catarina coast was more intense between 1789 and 1799, with the arrival of slaves coming from Pernambuco and Rio de Janeiro. African slaves and their descendants made up about 23.5% of the population of Santa Catarina from 1803 to 1857 [42].

In 1947, on the mainland area of Florianópolis, Malvina Ayroso de Barros, or Mother Malvina, founded the first terreiro of Umbanda, responsible for opening the way for the emergence of other rituals, such as Ritual de Almas e Angola in 1951, and the Candomblé that emerged in the region in the mid-1970s [44].

### Data collection and analysis

We conducted a survey of the existing terreiros on the Island of Santa Catarina, identified through the website of the Union of Black Culture of Santa Catarina [45], which provides contact with the terreiros of Afro-Brazilian religions registered in the region. This was complemented by online searches, resulting in a list of 40 terreiros. From the terreiros contacted, we used the snowball technique [46], in which the collaborators indicated other possible participants to be interviewed, each having the same characteristics (spiritual leaders of Candomblé, Umbanda, or Ritual de Almas e Angola, on the Island of Santa Catarina), thus covering as many people as possible fitting the research criteria.

We collected the ethnobotanical information through semi-structured interviews, between December 2015 and November 2016, related to the use of plants in the terreiros. We asked if plants were used as a healing (medicinal) resource, and if so, which plants and how were they used, as well as the socioeconomic information of the interviewees. Before data collection, we performed a pilot study in two terreiros outside the study area to adjust the methods and the interview protocol. The interviewees’ participation was conditional to a prior informed consent agreement.

We also collected information through participant observation and guided tours [47]. We used participant observation [46] to gain a deeper understanding of the religious context associated with the use of plants. The guided tours were carried out in the backyards of the terreiro properties and in forest areas located nearby the terreiros, with the purpose of collecting the plants for botanical identification. We collected botanical material and identified the plants following standard botanical collection procedures [48], and in accordance with the Biodiversity Information and Authorization System - SISBIO (Registration No: 49909 -1). We identified the botanical species using specific identification textbooks and expert consultations. The collected materials deposited in the Herbarium FLOR of the Federal University of Santa Catarina, and the Herbarium of the Agrotechnical School of Manaus (EAFM), of the Federal Institute of Education, Science, and Technology of Amazonas, under deposit numbers FLOR 60838 to 60857, EAFM 16715 to 16717, and EAFM 16744 to 16751. Plant names were checked with the plant list [49].

Some of the plant species cited in the interviews were not collected because the interviewee was unable to show them, often due to old age, or the absence or low abundance of specimens in backyards. Plants used in the rituals that were brought by the followers of the terreiro could not be collected. Whenever possible, we identified the non-collected plants based on the description of the plant made by the interviewee, the popular name, and with the aid of specific texts [50–53].

We analyzed the diseases through descriptive statistics. We categorized the diseases according to ICPC (International Classification of Primary Care) [54] as recommended by Weckerle et al. [55].

The similarity of knowledge was measured through an exploratory analysis of the cluster associated with the evaluation of the consistency of the clusters by SIMPROF analysis, due to the different number of terreiros between the religions. We used a matrix of presence and absence of the mentioned species. From this matrix, we calculated the Sorensen similarity matrix, with UPGMA clustering.

We considered those plant species with a higher percentage of citations in the three religions as culturally important plants. We performed a literature review of the ethnobotanical information of culturally important species by combining the term “ethnobotany” with “Afro-Brazilian” or “Candomblé” or “Umbanda” or “Almas e Angola.” We did not search for exhausting information of each species, but to show the existence of studies that show the use of these plants by other afro Brazilian groups.

We classified the plants concerning the place of origin according to the system suggested by Vavilov [56] based on eight centers of diversity or biogeographic regions: I—Chinese, II—Indian, III—Central Asian, IV—Near East, V—Mediterranean, VI—Ethiopian, VII—Mexican and Central American, VIII—South American. The classification was based on Prance and Nesbitt [57], Lorenzi and Matos [52], and complemented with Flora do Brasil 2020 [58].

To explore the adaptive process of maintenance and replacement in the use of plants [21], we analyzed the proportion of Neotropical and African components in relation to other places of origin, and in relation to liturgical and physical illness. The plant origins were grouped into three major groups: Neotropical (groups VII and VIII), African (VI), and Others (I to V). The differences between the plants of Neotropical and African origins in the context of liturgical or physical illness were compared through a chi-squared test. Only the plants mentioned exclusively for liturgical or physical illnesses (and not both uses) were incorporated into the analysis.

## Results

### Description of the collaborators

Among the 40 terreiros initially identified, 13 were excluded due to either: refusal to participate in the study, were not contactable, or were permanently closed. Twenty-seven *Babalorixás* and *Yalorixás* (Saint Mothers and Fathers, or spiritual leaders) were interviewed from Afro-Brazilian terreiros on the Island of Santa Catarina. Of those, 13 practiced the *Ritual de Almas e Angola*, 11 *Umbanda,* and 3 *Candomblé*. The group of 27 interviewees was composed of 9 men and 18 women, aged between 34 and 67 years (mean of 56.48 years, s.d. 8.77). Seventeen were retired and performed exclusively, the role of the spiritual leader, while ten affirmed that they worked in other activities besides performing the function of spiritual leader. Only two respondents did not reside in the same location as their terreiros, which were usually located behind their houses.

The studied terreiros were located in urban areas, with restricted space for the cultivation of plants. However, even within this limited space, we observed the cultivation of many useful plants as well as those collected from the wild, in nearby forests, where rituals are also performed.

### Knowledge and use of plants

We recorded 93 plants used in the terreiros, from which it was possible to identify 86 species belonging to 37 botanical families. The mean number of plants cited per respondent was 35.6 species (s.d. = 3.6). Of the cited species, 42% are exclusively for medicinal use and not linked to a liturgical use, 32% are indicated only for liturgical uses and 26% of the species are used in both contexts.

**Fig. 1:**
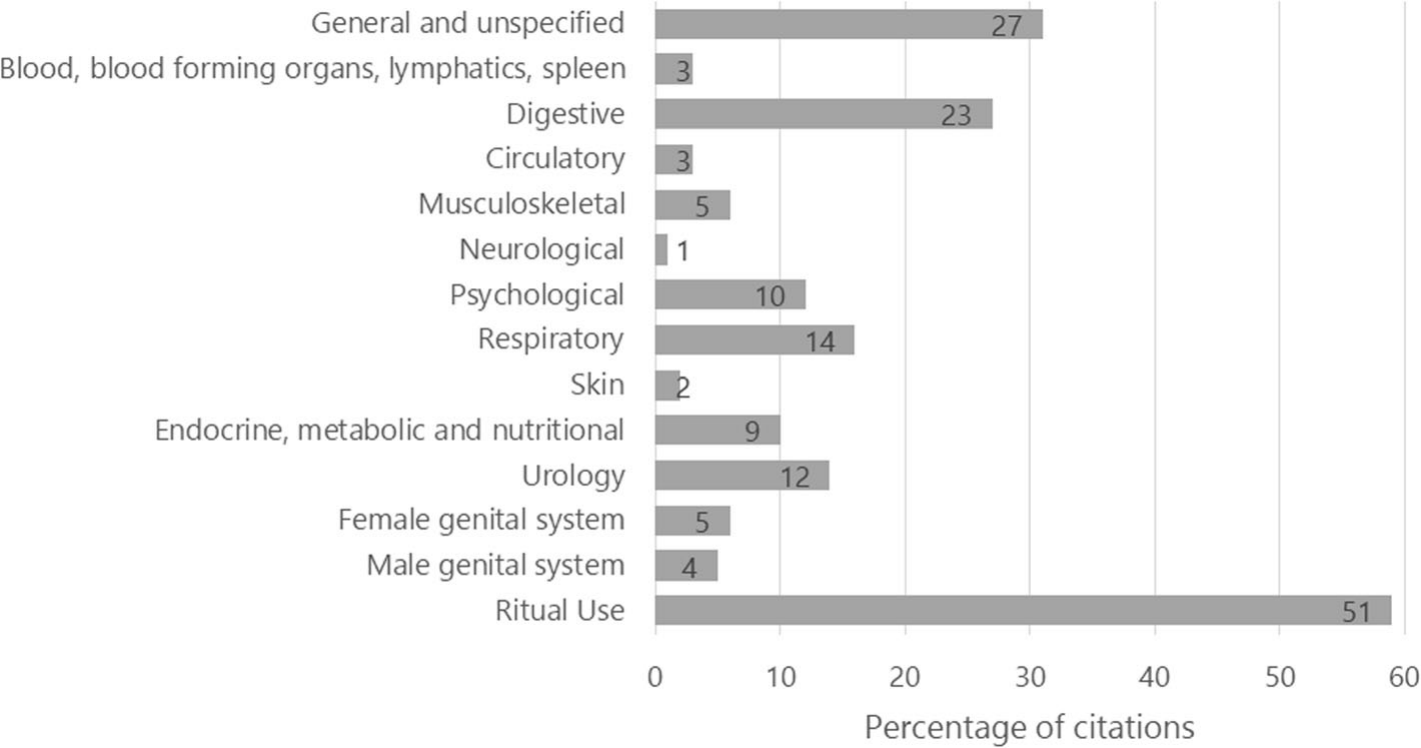
Indication of use of the 86 plants cited by the interviewees from 27 *terreiros* in the Island of Santa Catarina, Brazil. Absolute values are indicated inside each bar

We identified 14 categories of uses (Fig. 1), with emphasis on liturgical use (59% of the species), general and unspecified diseases (32%), digestive (27%), respiratory (15%), urological (14%) psychological (12%), and endocrine, nutritional and metabolic diseases (10%). We classified a plant as liturgical when the use was not directly related to a physical issue (e.g., cleansing and harmonizing baths, protection against the evil eye). However, we do understand that a liturgical use also has a medicinal and healing effect, and a medicinal use frequently involves some kind of ritual.

**Fig. 2:**
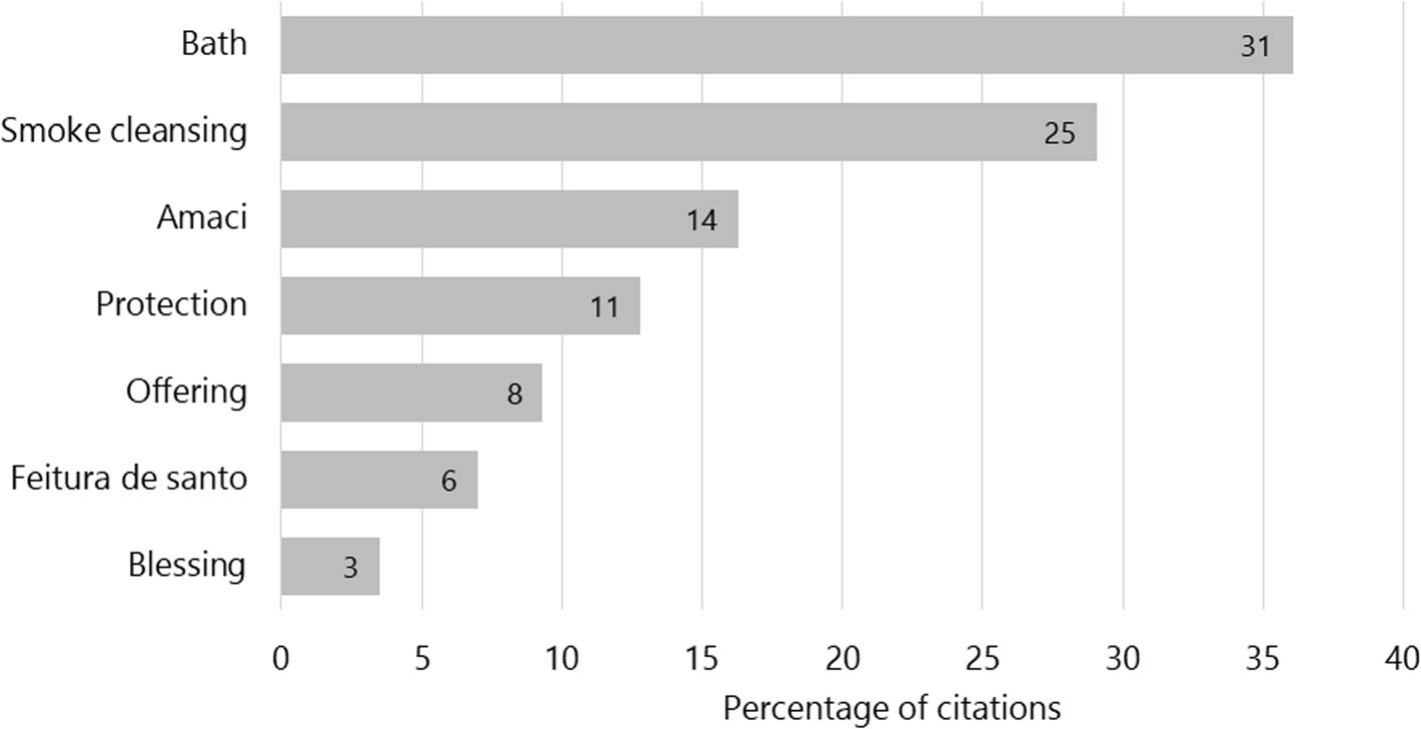
Uses of the 50 liturgical plants cited by interviewees from 27 *terreiros* in the Island of Santa Catarina, Brazil. Absolute values are indicated inside each bar

Among the species cited for use directly related to a physical issue, the most indicated forms of use were teas and infusions (54%) and antiseptic gargles (7%). Within the context of liturgical use, seven forms of use were identified: bathing, smoke cleansing, *amaci*, protection, offering, “making of saint” (or *feitura de santo*), and blessing. *Amaci* is a specific washing ritual. It occurs annually, in a day defined by each terreiro, and is a ritual of maintenance of the energies of already initiated people from the terreiro. In this ritual, several plants are mixed in a clay pot with water from the sea of waterfalls, and this water is used after a few days. The “making of saint” is an initiation ritual in which the person is kept recluse for a few days, and after that he/she is presented to his/her *Orixá* and to the plants guiding this entity, from which the bath is prepared. The most cited indications were for bathing, smoke cleansing, and *amaci*, with 35%, 29%, and 15% respectively (Fig. 2).

We recorded two types of baths: first, the energy cleansing or flushing, which aims to remove the dense energies of the patient, and second, the baths of harmonization or sweetness that are used to bring prosperity or happiness. The baths are prepared through infusion and are then poured over the body, allowing it to dry naturally. Different plants are used for each type of bath. In flushing baths, for example, stronger plants such as *arruda* (*Ruta graveolens*), *abre-caminho* (*Justicia gendarussa*), and *guiné* (*Petiveria alliacea*) are used. Whereas in the harmonization baths sweeter plants, like *alecrim* (*Rosmarinus officinalis*), *lavanda* (*Lavandula dentata*), and *menta* (*Mentha x villosa*) are used. Common baths can be indicated to initiated and non-initiated people, and usually the plant to be used is indicated by the priest, but not furnished to the user. Recommendations for plant collection are to collect the plants in the beginning on ending of the day (never at noon), and sometimes is also recommended to collect the plants during the full moon. Some priests recommend that women in their periods should not collect plants, to avoid the mixing of energies.

Smoke cleansing is also performed as a way to clean and harmonize people and spaces, through burning plants on charcoal. Many of the plants used in the cleansing baths are also used for smoke cleansing. Usually, the plants used for smoke cleansing are collected by one initiated person, who is also responsible for the drying process of the plants, and for the smoke cleansing itself. Smoke cleansing happens before the spiritual works, with the purpose to fill the gaps with the spiritual world and to better connect with spirits. The *amaci* is a ritual of washing the head of the mediums, using plants specific to each *Orixá*, aiming to connect the medium and strengthen the bonds with the spiritual guides.

The plants used for protection are those that have the power to block the negative energies of other people and from the self, as well as from the disembodied spirits. They are usually grown in pots or on the ground near socialization spaces. The *comigo-ninguém-pode* (*Dieffenbachia seguine*) is an example of a plant used for protection. The offerings or *ebó* are ritual offerings or animal sacrifices (which are not frequent and usually are of chicken, and rarely of goats) offered to the deities or ancestors, in which plants are also used. They are performed in appreciation of blessings received or seeking to resolve problems. *Pimenta-malagueta* (*Capsicum frutescens*), *Pimenta-dedo-de-moça* (*Capsicum baccatum*), and *Lírio-de-oxum* (*Hedychium coronarium*) are examples of plants used in offerings.

*Feitura de santo* (“making of saint”) is a term used for the ritual of initiation into the religion, in which the initiate happens to be officially a child of a saint (*filho de santo*) of the *terreiro*. The plants are placed for the initiate to lie on them, and in this way receive their vital energy (the *axé*). In the past, the initiated person had to lie on her/his plants during long time spans (up to 6 months), and frequently this required the person to move to the terreiro. Nowadays, this contact with the plant is done for 1 week or during one weekend. In the blessing, a branch of the plant is used, which the blesser holds in one hand and makes movements around the recipient with the other hand, being able to touch the recipient, or not. The blesser, however, is believed to be the spirit incorporated in the *medium*, and particularities of blessings vary between terreiros. Examples of plants used in blessings are common rue (*Ruta graveolens*), rosemary (*Rosmarinus officinalis*), and bellyache bush (*Jatropha gossypiifolia*).

The bath, the *amaci*, the smoke cleansing, and the *feitura de santo* are liturgical uses that have direct contact with the body. In this usage, the pharmacological principles of the plants may be acting. On the other hand, the plants used in offerings, and cultivated for protection, do not have direct contact with the physical body, and their use is associated with the magical and spiritual characteristics of these plants. The plants used in the blessings are considered to be of indirect use because there is little contact with the body of the individual being treated. Of the total number of plants mentioned, 93% are of direct use, including those used to cure specific physical issues (teas/infusions, gargles, syrups, among others) and the liturgical ritualistic ones (bathing, *amaci*, smoke cleansing, *feitura de santo*); 7% are plants of indirect use only (protection, offerings, and blessings).

The comparison between the plants used by the three religious groups showed that from the 86 plant species listed, 56 are used by all three groups, 20 are used only by *Ritual de Almas e Angola* and *Umbanda*, one species is used only by *Candomblé* and *Umbanda*, 2 species are exclusive to *Ritual de Almas e Angola* (*Artemisia absinthium* and *Datura suaveolens*), 1 exclusive to *Candomblé* (*Polyscias guilfoylei*), and 6 exclusive to *Umbanda* (*Pyrostegia venusta*, *Foeniculum vulgare*, *Fragaria* sp., *Peperomia pellucida*, *Vernonia condensata*, and *Pereskia aculeata*). Cluster analysis and SIMPROF showed no significant difference in knowledge about plants between groups, as they share a large number of species. There is a similarity of 89.41% between *Almas e Angola* and *Umbanda*; 70% between *Almas e Angola* and *Candomblé*, and 67.85% between *Candomblé* and *Umbanda* groups.

All respondents answered that they understood the plants as a basic and fundamental element for Afro-Brazilian religions, and without them, it would not be possible to carry out the religious works since the plants are holders of the axé, the primordial energy accessed in rituals.

### Culturally important plants

A set of sixteen plants (19% of all species identified) was cited by all the interviewees, demonstrating that although they are from different religious groups, much of the knowledge about the use of these plants is shared (Table 1). All the 16 shared species are used in the liturgical context, and seven of them are also used to heal issues related to specific physical symptoms. Two plants are only used as protection, without direct contact with the body: *Dieffenbachia seguine* and *Sansevieria cylindrica*. *Dieffenbachia seguine* is a toxic plant as are the two varieties of *Sansevieria trifasciata*, also used for protection, for rituals of bathing, *amaci,* and smoke cleansing.

**Table 1:** Plants cited by 27 interviewees from terreiros of *Ritual de Almas e Angola* (13 *terreiros*), *Candomblé* (3 *terreiros*), and *Umbanda* (11 *terreiros*) in Santa Catarina Island, Brazil

Local name	Voucher/collector number	Uses	Origin	Ethnobotany use Afro-Brazilian groups
**Acanthaceae**				
**Abre-caminho or quebra-demanda (*Justicia gendarussa*Burm. f.)**	FLOR60846	Energy cleaning (*amaci*, bath, smoke cleansing)	II	*Candomblé* and *Umbanda* [59]
**Araceae**				
**Comigo-ninguém-pode (*Dieffenbachia seguine*(Jacq.) Schott.)**	TP10	Evil eye (protection)	VIII	*Candomblé* and *Umbanda* [59–61], Maroons [6]
**Asparagaceae**				
**Espada-de-Santa-Bárbara or Espada-de-Iansã (*Sansevieria trifasciata*var.*laurentii*(De Wild.) N.E.Br, syn.*S. trifasciata*)**	TP06	Energy cleaning (*amaci*, bath, smoke cleansing); protection;*feitura de santo*	VI	*Umbanda Nagô* [62], *Candomblé* and *Umbanda* [59, 63]
**Espada-de-São-Jorge or Espada-de-ogum (*Sansevieria trifasciata*Prain)**	TP08	Energy cleaning (*amaci*, bathing, smoke cleansing); protection;*feitura de santo*	VI	*Umbanda Nagô* [62], *Candomblé* [60, 64], *Candomblé* and *Umbanda* [59, 61, 63], *Maroons* [6]
**Lança-de-Ogum (*Sansevieria cylindrica*Bojer ex Hook.)**	-	Evil eye (protection)	VI	*Umbanda Nagô* [62], *Candomblé* and *Umbanda* [59]
**Lamiaceae**				
**Alecrim (*Rosmarinus officinalis*L.)**	TP13	Energy harmonization (*amaci*, bath, smoke cleansing); feitura de santo; blessing;depression, poor digestion, cardiovascular problems (tea)	V	*Candomblé Kétu Nàgó* [65], *Candombl é*[64], *Candomblé* and *Umbanda* [61, 63], *Umbanda* [66, 67], African-based religions [68, 69], Afro-brazilian religions [3], Maroons [70–72]
**Alfazema (*Lavandula angustifolia*Mill.)**	TP59	Energy harmonization (bath, smoke cleansing);*feitura de santo*	V	*Candomblé* and *Umbanda* [61]
**Boldo-de-Oxalá (*Plectranthus barbatus*Andrews)**	FLOR 60855	Energy cleaning (amaci, smoke cleansing, bath), offering; bronchitis (syrup)	II	Afro-Brazilian religions [3], *Candomblé* [64], Maroons [6, 70, 72]
**Hortelã (*Mentha x villosa*Huds.)**	TP17	Energy harmonization (bath); soothing (tea)	V	*Umbanda Nagô* [62], *Candomblé* and *Umbanda* [61, 63], *Umbanda* [66], Maroons [70, 72]
**Lavanda (*Lavandula dentata*L.)**	FLOR 60854	Energy harmonization (bath, smoke cleansing)	V	Umbanda [67]
**Manjericão (*Ocimum basilicum*L.)**	FLOR 60841	Energy cleaning (smoke cleansing, bath); feitura de santo; offering;bronchitis (tea, syrup)	II	*Candomblé Kétu Nàgó* [65], *Candomblé* and *Umbanda* [59], Umbanda [67], African-based religions [69], Afro-Brazilian communities [73], Afro-Brazilian religions [3], Maroons [6, 70, 71]
**Phytolaccaceae**				
**Guiné (*Petiveria alliacea*L.)**	FLOR60856	Energy cleaning (amaci, bath, smoke cleansing); protection	VIII	*Candomblé* [2, 74], *Umbanda* [66, 67], African-based religions [68, 69], Afro-brazilian religions [3], Maroons [75]
**Rutaceae**				
**Arruda (*Ruta graveolens*L.)**	TP11	Energy cleansing (*amaci*, bath, smoke cleansing); blessing; protection; *feitura de santo*	V	R: *Umbanda Nag ô*[62]*, Candomblé* [64, 74], *Candomblé* and *Umbanda* [59, 61, 63],*Umband a*[66, 67], Afro-Brazilian religions [3], Maroons [6, 70–72, 75]
**Solanaceae**				
**Fumo (*Nicotiana tabacum*L.)**	-	Energy cleaning (*smoking*)	VIII	*Candomblé* and *Umbanda* [61, 63], African-based religions [69]
**Pimenta- malagueta (*Capsicum frutescens*L.)**	TP16	Offering; baths in specific cases of protection; for lack of energy, anti-inflammatory, aphrodisiac, antiarthritic, anticancer	VIII	*Umbanda Nagô* [62], Maroons [6]
**Pimenta-dedo-de-moça (*Capsicum baccatum*L.)**	TP15		VIII	*Umbanda Nagô* [62]

Origins: II, Indian; V, Mediterranean; VI, Ethiopian; VII, Mexico and Central America; VIII, South American

All culturally important plants were recorded in other ethnobotanical studies with Afro-Brazilian groups (Table 1). *R. officinalis*, *O. basilicum*, *R. graveolens,* and *P. alliacea* are the plants with the largest number of records, demonstrating their wide use in the various Afro-Brazilian regions and groups, as well as in different regions of Brazil.

Eight of these culturally important plants originate from the Old World (five from the Mediterranean region and three from India), which may reflect the ethnobotanical knowledge exchange and adaptation of ritualistic plants from contact between the African diaspora and European settlers. Besides, five of these plants originate in the Neotropical (South and Central America), which may indicate the exchange and integration of rituals and knowledge of local indigenous peoples. Only three of these plants, the species of the genus *Sansevieria*, originate from the African continent, whereas *Sansevieria trifasciata* is native to tropical West Africa from Nigeria east to the Congo and *S. cylindrica* is native from Angola.

### Neotropical and African components

**Fig. 3:**
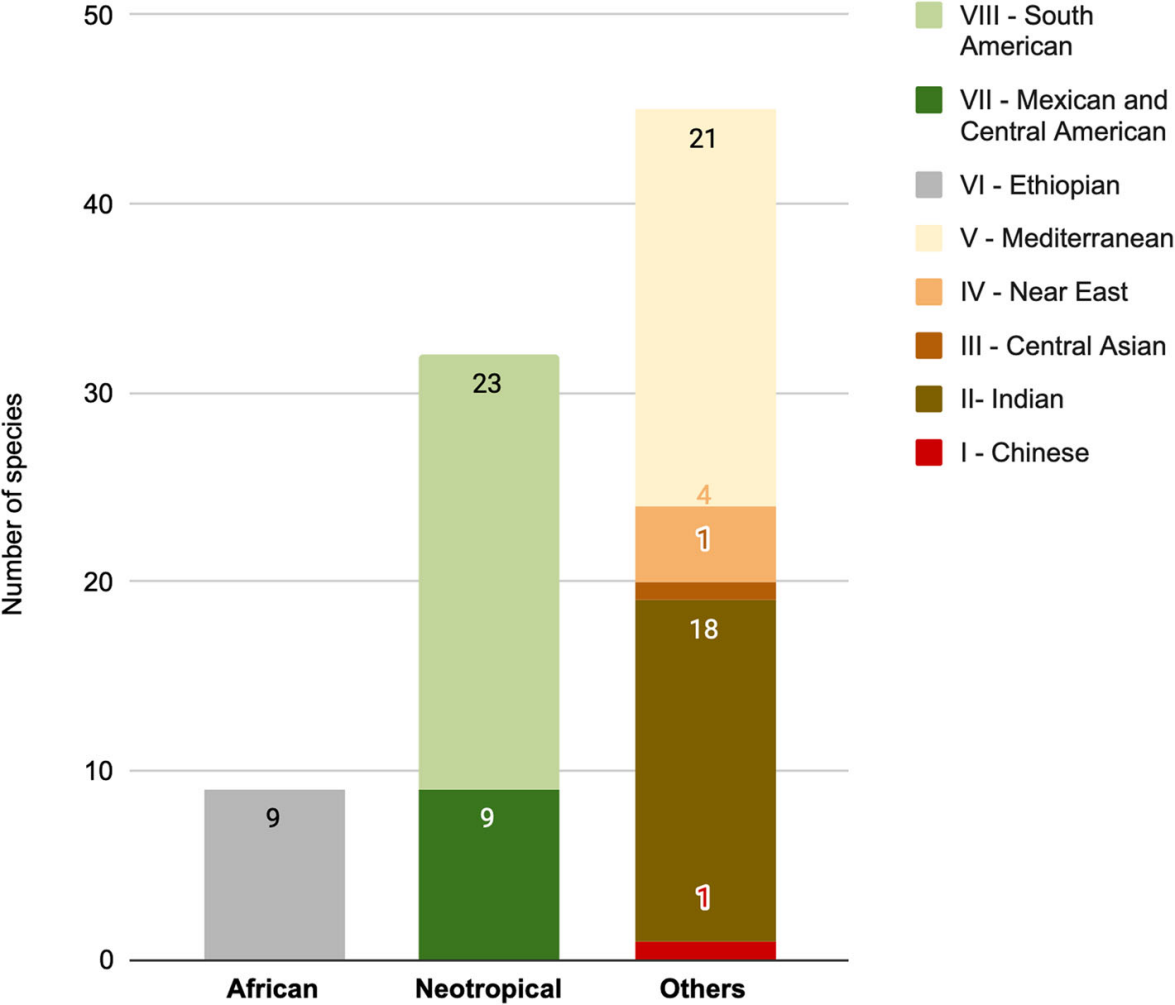
Geographic origin of the 86 plants cited by interviewees from 27 terreiros in the Island of Santa Catarina, Brazil. Absolute values are indicated inside each bar

Most of the plants are from other regions than home (African) and host (Neotropical) migrants continents (Fig. 3). Among the plants of African origin (9 species), only one is associated exclusively with physical illnesses, 5 are exclusive to liturgical uses and 3 are used for both, showing some preference in maintaining the use of plants from the continent of origin for ritual and liturgical uses. Plants of other origins are used equally for ritualistic use as well as to treat physical problems. Among the plants of Neotropical origin, 17 are used exclusively to deal with physical issues, 9 exclusively for liturgical rituals, and 6 for both uses. For the plants of other origins, 15 are exclusively used to address physical illnesses, 14 for liturgical uses, and 16 for both. Chi-square comparison between plants of African and Neotropical origins for liturgical uses and body problems was not significant (chi-square with Yates correction = 2.9304, *p* < 0.05). However, the proportion of Neotropical: African plants of 1.8 for ritualistic and 17 for body or physical illnesses, that means that for each 1.8 Neotropical plant cited for liturgic use, 1 African plant is mentioned; but for every 17 plants for physical illness use of Neotropical origin, 1 African plant is cited. These results emphasize that the process of maintenance of African plants is stronger in the context of liturgical rituals than for specific physical issues. In the context of replacement, both Neotropical components and from other origins played an important role, since both were incorporated through exchange with other cultures in the ritualistic and physical contexts.

## Discussion

The richness of plants registered in the *Candomblé*, *Umbanda,* and *Almas e Angola* religions on Santa Catarina Island reinforces the importance of plants in Afro-Brazilian religions, in the conceptualization of health, and the religious and cultural identity of these groups. Plants are an essential element for these Afro-Brazilian religions because the plants are detainees of the primordial energy accessed in rituals—axé. Even with the terreiros being located in urban areas, where there are few spaces for the collection and cultivation of plants, these areas still maintain the use of a diversity of plants.

In general, the three religions presented a highly shared knowledge base (sharing 65% of the species), which may reflect both the shared origin of these religions and a possible contemporary exchange of information between the interviewees. *Umbanda* and *Ritual Almas e Angola* presented a greater number of shared species, which may be related to the origin of *Almas* e *Angola* as a branch of *Umbanda*, but can also be influenced by the smaller sample size of *Candomblé terreiros* (only 3 terreiros). The few species unique to each religion can reflect distinctive inner customs and traditions within groups, such as specific initiation practices, offerings, and obligations. However, we observed high variability in the specific practices of each *terreiro*. Although the spiritual beings play a very central role in these religions, it would be interesting if other studies investigated the process of transmission and adaptation of this knowledge, and cultural resistance in the Afro-Brazilian religions.

A highlight in this study is the existence of a group of sixteen plants that was shared by 100% of the interviewees. While much knowledge may be considered diffuse knowledge, which is, shared by many communities, it is rare in ethnobotanical studies for knowledge of a given set of plants to be shared by all or most of the respondents. This high degree of shared knowledge among interviewees may be reflecting a capacity for cultural resilience, and unity among these religious groups. A few studies on medicinal plants in groups of Afro-descendants, citing plants used by the collaborators, showed that the shared knowledge base (the same species mentioned by different interviewees) is usually lesser than 50% of the collaborators [71, 72], or with few species with a quota above 50% [76].

Also, these sixteen plant species shared by all terreiros must possess a cultural value in the formation of the local identity of these religious groups. The sixteen plants shared by all interviewees appear in other studies with Afro-Brazilian groups, demonstrating its importance to Afro-Brazilian identity, local health systems and religious rituals, independent of the Brazilian region [2, 3, 59–70, 74]. The cultural importance of some species is not restricted to Afro-Brazilian groups, since some plants are common in Brazilian pharmacopoeias, such as *Rosmarinus officinalis, Plectranthus barbatus*, and *Ruta graveolens* [52]. Some of them are also used in Afro-religious contexts in other places in South America, such as Suriname, and in African countries such as Gabon and Benin (e.g., species of the genera *Justicia*, *Ocimum*, and *Capsicum* [77, 78]. Thus, this consensual set of 16 plants may be one of the links that connect them to the common African ancestral origin within this Brazilian historical and social context.

Plants used to heal specific physical conditions are used in the terreiros mainly to treat simple health problems, such as digestive problems (e.g., stomach aches and nausea), and general and unspecified diseases (e.g., pain and inflammation in general), and this what was also registered in studies in the context of Afro Brazilian religions [3]; with Maroons [6] and with medicinal/magic plants from a public market [79]. On the other hand, it is important to emphasize that these categories of use are not restricted to Afro-Brazilian groups, and were also observed in studies on the complementary use of medicinal plants and biomedicine [80], showing the preference of using plants to treat simpler health problems such as digestive, respiratory, and general pains. A fact that should be highlighted is the large number of species indicated for ritual use related to the magic-religious nature of the three groups, confirming other studies of plants of African-Brazilian rituals [8, 63, 64, 68].

Among the liturgic ritual use, those with direct contact with the body, such as bath, smoke cleansing, *amaci* and *feitura de santo*, stand out. Baths also had the highest indications for ritual use in similar studies by Carvalho et al. [61], Gomes et al. [63], and Pires et al. [64], as well as in studies in Suriname with African descendants [81] and African peoples of Benin and Gabon [23]. These forms of use highlight the maintenance and adaptation of imported cultural practices for African descendants throughout the diaspora process [2]. In addition, the *amaci* can also be considered as a specific form of bath, directed only to the “sons/daughters of saint” (*filhos de santo*), but in this work we treated them separately from the baths because this is how the Saint Mothers and Fathers referred to them.

Species used for bath, smoke cleansing, *amaci*, and *feitura de santo* were reported for similar purposes in other studies, such as the use for baths of *Justicia gendarussa* and *Sansevieria* spp. in Minas Gerais (Brazilian Southeast) [59], and *Lavandula dentata* in other parts of southern Brazil [65]. These and other species with ritualistic uses were investigated for pharmacological properties, such as *J. gendarussa* [82, 83], *S. trifasciata* [84, 85], *L. angustifolia* [86], *L. dentata* [87], *R. graveolens* [88]. However, except for *Petiveria alliacea*, none of the 16 plants had these properties investigated in the context of the ritualistic use. After simulating the route of administration of the site where the ritual is performed, Alves et al. [89] found no effect on locomotor activity or anxiety-like behavior of *P. alliacea* in laboratory animals, and called the attention to possible negative effects of such administration.

These ritualistic uses of direct contact with the body may indicate that the use of plants in rituals is based not only on the magical power of plants but also on the medicinal principles of these plants, as already evidenced by Quiroz et al. [23] and Garcia et al. [67]. Also, it is important to emphasize that the ritualistic use of indirect contact with the body (e.g., offerings and protection) does not indicate that they have no action on people’s health. The health system associated with Afro-Brazilian religions, as well as traditional health practices, are based and justified within their culture of existence.

The high number of plants from other places like the Mediterranean and Asia Minor regions is related to the diversity of species that were introduced to Brazil by the colonizers that were subsequently incorporated by the Africans as a substitute for medical and ritualistic practices in the new continent [3]. In this way, the process of construction of the cultural identity of these Afro-Brazilian religious groups involved the participation and contribution of many cultures, in a process of appropriation, import, and export of species [1, 2, 11]. The maintenance of a few species from the Ethiopian region (continent of origin), such as the *Sansevieria* plants, may seem contradictory because they are Afro-Brazilian religions, but this may be related to the difficulty that slaves had in bringing their plants on the slave ships, as discussed in the works of Voeks [9, 10] and Matory [90]. On the other hand, the maintenance of plants of African origin associated with liturgical use reinforces the importance of these religions in keeping their identity and of cultural strengthening of these groups. Also, the presence of Neotropical plants illustrated by species such as *Dieffenbachia seguine*, *Petiveria alliacea*, *Nicotiana tabacum*, and plants of the genus *Capsicum* may be a result of the exchange of ethnobotanical knowledge between indigenous people and people of African descend [9], leading to current Afro-Brazilian pharmacopoeias being numerically dominated by Neotropical or Mediterranean taxa. In addition, it is important to highlight that *P. alliacea* (a Neotropical plant) is employed in West Africa and Brazil for similar medicinal and magical purposes [91], demonstrating that the exchange of knowledge also influences the medicinal repertoire in the continent of origin of these groups.

All 16 culturally important species are used in liturgic rituals, and half of them are also used to treat physical issues. Toxic plants are mainly used for protection, and this may be related directly to the knowledge of the toxic effects of these plants held by traditional people. In the case of *D. seguine*, its magical use is believed to have been built from its use as a toxic plant to punish slaves [92].

The wide use of these plants by different Afro-Brazilian groups may be an indicator that this use is not only due to the magical properties of the plant, as previously discussed, especially in the cases in which the mode of the application allows some contact of the person to be healed with the plant used. Also, these results highlight the importance of promoting phytochemical and pharmacological investigations into species of ritual use to understand the effect of their external application on the human body [23, 67].

As well as calling attention to the pharmacological potential of these species, we also highlight the importance of the ethical issues associated with ethnobotanical and pharmacological research. It is fundamental to value and respect the people who have for so long created, innovated, and transmitted such knowledge that benefits so many people, including in contemporary urban societies, benefiting from drugs derived from traditional knowledge. To achieve this, researchers must develop their research following the international guidelines of the Convention on Biological Diversity, as well as any specific national legislations and regulations, for the protection of this local knowledge and benefit-sharing. In this way, we collaborate to reduce the prejudice and invisibility experienced by groups of Afro-Brazilian religions in Brazil, and in other American countries.

As discussed by Quiroz et al. [23] and Reyes-García [93], the results of this study also make us reflect on our Western views on the ritualistic use of plants, which are often treated only as a matter of “belief and faith.” This view stems from Western science’s tendency to separate medicine from spirituality and religion and the limitations we often have on recognizing and values traditional knowledge and practices in Western science. In this way, we hope that the results of this study can contribute to the valorization of these ritualistic practices. The industrialized and urban society has benefited from the knowledge of the ancestral people through centuries of exploitation and now is the time to build a new form of relationship with the knowledge of these people, with respect and recognition.

Finally, it is also important to recognize some limitations of this study, such as the small number of *Candomblé terreiros*, which made it impossible to perform comparative statistical analysis among the three groups. Moreover, since the research dealt with knowledge considered sacred (and often secret) within the cultural and religious context, we assume that some information has not been shared with the researchers. Thus, the data presented in this study comprises only a part, of the broad and complex relationship of these religious groups with plants.

## Conclusions

The Afro-Brazilian religions of the Island of Santa Catarina use a diversity of plants to treat physical and spiritual issues. The plant uses to heal physical symptoms are mainly performed to treat simpler diseases, such as digestive problems, colds, and flu. Concerning liturgical ritual use, in most situations, the direct contact of the plants with the body, as in the case of baths and smoke cleansing, indicates that the active principles of plants may also be having an effect. A group of 16 plants was shared by all the collaborators, demonstrating the importance of these species for the cultural and religious identity of these Afro-Brazilian groups on the Island of Santa Catarina. The presence of these plants in other studies of Afro-Brazilian religions in other parts of Brazil stands out as a characteristic widely shared of these practices, regardless of regional particularities of history and environment. Recognizing the importance and breadth of modern medicine, and even considering the advances in incorporating therapeutic practices that are not only based on drugs, but there is still room for the recognition and appreciation of knowledge and practices of Afro-Brazilian religions, which are constantly undergoing a process of prejudice and discrimination. Thus, we emphasize the importance of recognizing and valuing this ancestral Afro-Brazilian knowledge and learning also from these people about their broader vision of health, combining material and non-material components, which also adds more spirituality in health care.

## Data Availability

The datasets used and/or analyzed during the current study are available from the corresponding author on reasonable request.
